# Ketogenic diet and fasting diet as Nutritional Approaches in Multiple Sclerosis (NAMS): protocol of a randomized controlled study

**DOI:** 10.1186/s13063-019-3928-9

**Published:** 2020-01-02

**Authors:** Lina Samira Bahr, Markus Bock, Daniela Liebscher, Judith Bellmann-Strobl, Liane Franz, Alexandra Prüß, Dania Schumann, Sophie K. Piper, Christian S. Kessler, Nico Steckhan, Andreas Michalsen, Friedemann Paul, Anja Mähler

**Affiliations:** 1 NeuroCure Clinical Research Center and Department of Neurology, Charité—Universitätsmedizin Berlin, corporate member of Freie Universität Berlin, Humboldt Universität Berlin, Berlin, Germany; 2 Institute of Biochemistry, University Medicine Berlin—Charité, Charitéplatz 1, D-10117 Berlin, Germany; 3 Department of Medicine B, Ruppin General Hospital, Brandenburg Medical School, 16816 Neuruppin, Germany; 4 Department of Hand Surgery, Upper Extremity and Foot Surgery, Center for Orthopedics and Trauma Surgery, Hospital Waldfriede, Argentinische Allee 40, 14163 Berlin, Germany; 5 Institute of Social Medicine, Epidemiology & Health Economics, Charité—Universitätsmedizin Berlin, Berlin, Germany; 6 Experimental and Clinical Research Center, a cooperation between Charité—Universitätsmedizin Berlin and Max Delbruck Center for Molecular Medicine, Berlin, Germany; 7 Berlin Institute of Health, Berlin, Germany; 8 Institute of Biometry and Clinical Epidemiology, Charité—Universitätsmedizin Berlin, corporate member of Freie Universität Berlin, Humboldt-Universität zu Berlin, and Berlin Institute of Health, Charitéplatz 1, D-10117 Berlin, Germany; 9 Department of Internal and Integrative Medicine, Immanuel Krankenhaus Berlin, Berlin, Germany; 10 DZHK (German Centre for Cardiovascular Research), Berlin, Germany

**Keywords:** Multiple sclerosis, Dietary intervention, Ketogenic diet, Intermittent fasting, Anti-inflammatory diet

## Abstract

**Background:**

Multiple sclerosis (MS) is the most common inflammatory disease of the central nervous system in young adults that may lead to progressive disability. Since pharmacological treatments may have substantial side effects, there is a need for complementary treatment options such as specific dietary approaches. Ketone bodies that are produced during fasting diets (FDs) and ketogenic diets (KDs) are an alternative and presumably more efficient energy source for the brain. Studies on mice with experimental autoimmune encephalomyelitis showed beneficial effects of KDs and FDs on disease progression, disability, cognition and inflammatory markers. However, clinical evidence on these diets is scarce. In the clinical study protocol presented here, we investigate whether a KD and a FD are superior to a standard diet (SD) in terms of therapeutic effects and disease progression.

**Methods:**

This study is a single-center, randomized, controlled, parallel-group study. One hundred and eleven patients with relapsing–remitting MS with current disease activity and stable immunomodulatory therapy or no disease-modifying therapy will be randomized to one of three 18-month dietary interventions: a KD with a restricted carbohydrate intake of 20–40 g/day; a FD with a 7-day fast every 6 months and 14-h daily intermittent fasting in between; and a fat-modified SD as recommended by the German Nutrition Society. The primary outcome measure is the number of new T2-weighted MRI lesions after 18 months. Secondary endpoints are safety, changes in relapse rate, disability progression, fatigue, depression, cognition, quality of life, changes of gut microbiome as well as markers of inflammation, oxidative stress and autophagy. Safety and feasibility will also be assessed.

**Discussion:**

Preclinical data suggest that a KD and a FD may modulate immunity, reduce disease severity and promote remyelination in the mouse model of MS. However, clinical evidence is lacking. This study is the first clinical study investigating the effects of a KD and a FD on disease progression of MS.

**Trial registration:**

ClinicalTrials.gov, NCT03508414. Retrospectively registered on 25 April 2018.

## Introduction

Multiple sclerosis (MS) is the most common chronic inflammatory autoimmune disease of the central nervous system. It leads to neurodegeneration and is a major cause of progressive disability and early retirement in young adults [1–3]. According to estimates, more than 2 million people worldwide are affected [4]. Relapsing–remitting MS (RRMS) is the most common form of MS and is characterized by relapses and periods of remission as well as systemic inflammation of the central nervous system and progressive neurodegeneration from the earliest disease stages [5, 6].

Currently, there is no cure for MS, but several immunomodulatory therapies are available that may slow disease progression [7]. However, all of them may have substantial side effects and patients respond differently due to the complex nature of the disease. Consequently, there is a need for complementary therapies such as specific diets that may reduce MS symptoms, improve the patient’s quality of life and even delay disease progression. A recent review reported that up to 70% of MS patients already use alternative and complementary medicine [8].

It is widely accepted that a combination of genetic susceptibility and environmental factors causes MS [9]. That nutrition might be among these factors is supported by the fact that MS is more frequent in western countries than in less-developed nations [10]. The so-called western diet is high in energy, saturated fats and sugars. Of note, a meal high in fat and refined carbohydrates has been shown to cause a more pronounced postprandial insulin secretion and enhanced inflammation compared to a meal high in fiber and fruits in healthy lean subjects [11]. In line with this, a 12-week ketogenic diet (KD) decreased insulin resistance and several serum inflammatory markers in overweight men and women [12].

These are important findings since insulin resistance seems to be more common in MS patients than in healthy controls and is associated with higher Expanded Disability Status Scale (EDSS) scores [13, 14]. Furthermore, elevated serum levels of the pro-inflammatory cytokine IL-17 in MS patients [14] may contribute to the impaired glucose and insulin metabolism in MS [15].

The cerebral glucose hypometabolism that occurs in MS patients [16–18] is thought to reflect mitochondrial dysfunction in neuronal cells [19]. Although the mechanism is not yet defined, it is feasible that neurodegenerative processes driven partly by oxidative stress contribute to this mitochondrial dysfunction [20]. Indeed, several studies have demonstrated increased markers of oxidative stress and decreased compensatory antioxidative capacity in MS patients [14, 21–23].

Profound reduction of either overall calorie or carbohydrate intake decreases glucose and insulin levels, thereby driving the body to produce ketone bodies from internal or external fats. These ketone bodies provide an alternative energy source for the brain. They might even be more efficient (per unit oxygen) than glucose [24]. Furthermore, ketone bodies seem to stimulate mitochondrial biogenesis and reduce mitochondrial permeability [25, 26].

KDs and fasting diets (FDs) both drastically reduce carbohydrate intake and it has been suggested that the resulting ketone bodies facilitate the regeneration of demyelinated axons [20]. It is thus feasible that either of these dietary approaches could have a therapeutic benefit in MS patients.

Fasting not only induces ketosis, but might also activate autophagy in the brain, liver and muscle [27]. Macroautophagy is a key quality-control pathway in cells through which nonnuclear parts of the cell and cytoplasmic macromolecules are renewed and mobilized, respectively. It is thought to mediate the anti-aging effects of calorie restriction [28].

Since some of the effects of fasting can be replicated with a KD, autophagy might also be relevant for KDs. Indeed, current evidence from a rat model suggests that a KD can attenuate seizure-induced neuronal injury via autophagy [29].

The KD was originally conceptualized to mimic the biochemical effects associated with fasting [30] and was used as an alternative treatment in pharmaco-resistant childhood epilepsy as early as the 1920s [31]. Since then, KDs have also been shown to improve symptoms in other neurodegenerative diseases such as Alzheimer’s disease and Parkinson’s disease [32, 33].

In the case of MS, there is preclinical evidence for the efficacy of KDs and FDs from experimental autoimmune encephalomyelitis (EAE), the established animal model of MS. A KD slowed disease progression, improved motor disability and hippocampal atrophy, reversed lesions and suppressed inflammatory cytokines and reactive oxygen species [34]. A fasting mimicking diet was shown to delay onset and slow disease progression. This was accompanied by increased corticosterone levels, autoreactive lymphocyte apoptosis and oligodendrocyte regeneration during the fasting [35]. These findings are supported by other EAE studies showing beneficial effects of intermittent fasting and chronic calorie restriction [36–38].

Changes in the gut microbiome are associated with many disease states, including autoimmune diseases such as MS, and the gut microbiome is intricately linked to our immune system and inflammatory responses. The typical western diet is associated with gut microbial imbalance (dysbiosis), low-grade inflammation and neuroinflammation [39]. Interestingly, recent studies have shown that gut dysbiosis can also occur in MS patients [40–42]. Both KDs and FDs may positively affect the gut microbiota by enhancing gut microbial diversity [42, 43]. Thus, changes of the gut microbiome could be implicated in the effects of our dietary regimens.

We have already obtained clinical evidence for the feasibility and safety of KDs and FDs in MS patients and showed that they might improve health-related quality of life [35]. Another more recent pilot study which tested the safety and tolerability of a modified Atkins diet found decreased body mass index, body fat mass, fatigue and depression scores in 20 RRMS patients. This was, however, an uncontrolled study with just a 6-month intervention period, which cannot corroborate effects on disease progression [44].

Here, we present the protocol of a randomized, controlled clinical study investigating the effects of a KD and a FD compared to a standard diet (SD) in RRMS. The SD is a healthy, predominantly vegetarian diet that follows the recommendations of the German Nutrition Society (DGE). MS patients adhering to a similar diet for 12 months showed improved body mass index, metabolic markers and fatigue but no changes in brain MRI outcomes [45].

Thus, we hypothesize that KDs and FDs are superior to the SD with respect to new lesions on cranial T2-weighted MRI after 18 months compared to baseline. We further hypothesize differential effects of our interventions in men and women, because of sex-specific metabolic differences (e.g. fat and carbohydrate metabolism). We further expect larger treatment effects in patients without MS medication.

Secondary endpoints are safety aspects of the dietary interventions, brain volume, annualized relapse rate, disability progression, changes in gut microbiome, metabolomics of stool and blood, immunological effects, cognition, fatigue, depression, muscle strength, walking endurance and quality of life.

## Methods

### Study design

This is a single-center, randomized, controlled, three-armed, parallel-group study conducted at the NeuroCure Clinical Research Center at Charité—Universitätsmedizin Berlin. Recruitment started in April 2017 (ClinicalTrials.gov identifier: NCT03508414). Patients are recruited from all over Germany. Recruitment strategies include specific study calls on the website of the German Multiple Sclerosis Society and the distribution of study flyers in specialized neurological practices as well as information events and lectures for patients. We intend to randomize 111 RRMS patients to one of three 18-month dietary interventions: a KD with a carbohydrate intake restricted to 20–40 g/day; a FD with a 7-day fast every 6 months and 14-h daily intermittent fasting in between; and a SD that is predominantly vegetarian and fat-modified as recommended by the German Nutrition Society. The institutional review board of Charité—Universitätsmedizin Berlin approved the study and written informed consent is obtained from all participants prior to study entry (Additional files 1 and 2). The study is conducted in accordance with the Declaration of Helsinki in its currently applicable version, the guidelines of the International Conference on Harmonization of Good Clinical Practice (ICH-GCP) and applicable German laws. For further details refer to the SPIRIT checklist (Additional file 3).

### Participants

Patient information and informed consent have been prepared in accordance with the guidelines of the institutional review board of Charité—Universitätsmedizin Berlin. Potential participants receive both forms at least 24 h before a consultation with a study physician who personally explains all study procedures. If he or she is willing to participate and had sufficient time to ask questions, written informed consent is given. Afterward, inclusion and exclusion criteria are assessed. The main inclusion criteria are a definite diagnosis of RRMS according to the 2017 McDonald criteria [46], a stable immunomodulatory therapy or no disease-modifying therapy for at least 6 months, an EDSS score below 4.5 [47] and disease activity within the last 2 years before study entry. This is defined as at least one new lesion on brain MRI and/or at least one relapse. Participants are recruited in cohorts of 15–25 patients.

#### Complete list of inclusion criteria

- Relapsing–remitting MS
- 18–65 years of age
- EDSS < 4.5
- Stable immunomodulatory therapy or no disease-modifying therapy ≥ 6 months before study entry
- ≥ 1 relapse or ≥ 1 new T2 lesion or ≥ 1 contrast-enhancing lesion on MRI within the last 2 years
- Agreement that possible incidental findings will be communicated
- Body mass index between 19 and 45 kg/m^2^
- Ability to give verbal and written consent
- Health insurance

#### Complete list of exclusion criteria

- Start or change of immunomodulatory therapy < 6 months before or during the study
- Relapse or cortisone treatment within 30 days before study entry
- Clinically relevant metabolic, progressive or malignant diseases
- Intake of > 1 g/day omega-3 fatty acid supplements
- Significant cognitive-cooperative impairment
- Insulin-dependent diabetes mellitus (type I)
- Participation in another interventional study
- Weight loss diet or loss of more than 5 kg within 2 months before study entry
- Insufficient mental ability for cooperation
- Eating disorders
- Kidney stones
- Therapy with oral anticoagulants
- Pregnancy and breastfeeding
- Suspected lack of compliance
- Smokers (> 5 cigarettes per day)
- Known alcohol and drug abuse
- Inability to give informed consent or apply to the study protocol
- Contraindications for MRI such as metallic implants, cardiac pacemakers and claustrophobia

Retention of patients is promoted by close and frequent contact with their nutritional counselor and study physician via telephone, email and study visits. In case of relevant issues, visits outside the study schedule are offered.

Discontinuation criteria are withdrawal of consent, subsequent occurrence of an exclusion criterion (e.g. change of immunomodulatory therapy), lack of compliance and medical reasons for stopping the intervention.

For all three groups, compliance is defined as attending at least 8 of 10 group sessions and each of six study visits. Food records measure compliance to the prescribed intervention. In the KD group, the majority of blood ketone measurements should be ≥ 0.5 mmol/L. For the FD group, there are regular additional meetings during each of the three fasting periods. These meetings have to be attended, enabling the physician and the counselor to evaluate compliance.

We offer patients who meet a discontinuation criterion to attend the remaining study visits for follow-up outside the study protocol. We assess all outcomes except for exploratory blood parameters (autophagy, oxidative stress) in these patients.

### Randomization

Eligibility of patients will be determined at the screening visit by a trained physician. Afterward, randomization is done in three strata to distribute possible confounding factors equally to the dietary interventions. Strata are sex (man or woman), MS medication (yes or no) and lesion load (< 15 or ≥ 15 lesions) in the baseline brain MRI scan. We use a stratified block randomization with variable block length. This will ensure a homogeneous distribution of interventions over these strata. An external statistician who is not involved in the study carries out the randomization according to predefined randomization lists.

### Dietary interventions

Nutritional counseling for all three interventions takes place in small groups within 10 sessions over 18 months (five sessions in months 1–3, five sessions in months 4–18). The group setting facilitates exchange between patients, management of dietary challenges, monitoring of compliance and recording of adverse events. Between the group sessions, patients have the possibility to contact their nutritional counselor via email or telephone at any time.

- *KD*. During nutritional counseling, patients are instructed to start by limiting carbohydrate intake to just 20 g/day for 4 weeks in order to establish ketosis. Then, patients increase their carbohydrate intake by 5 g each week until they reach their individual maximum (approximately 40 g) to maintain stable ketosis. All carbohydrates relevant for elevating blood glucose are limited to 40–50 g/day. In addition, the glycemic index and glycemic load of carbohydrates have to be below 50 and 6, respectively. This ketogenic diet is equivalent to a traditional ketogenic diet, but with a liberalized macronutrient composition of 70–80% fats, 15–20% proteins and 5–10% carbohydrates (compared to a traditional ketogenic diet with 90% fat, 6% proteins and 4% carbohydrates). To aid in the adjustment and determine their individual carbohydrate intake limits, patients receive a hand-held ketone-meter to measure and record blood concentrations of ketone body β-hydroxybutyrate at regular intervals. Values should be between 0.5 and 3.0 mmol/L.
- *FD*. Patients in the FD group fast for 7 days every 6 months. During this intensive fasting episode, patients only consume vegetable juices and vegetable broth, yielding a daily calorie intake between 200 and 350 kcal. Tea and water is available *ad libitum* to ensure sufficient fluid intake. Two days before and 3 days after fasting, patients eat a low-calorie vegetarian diet for preparation and aftercare. The fasting is initialized by an intestinal clearing with laxatives (e.g. Glauber’s Salt by FX-Passage). Outside these intensive fasting episodes, patients intermittently fast for 14 h daily on 7 days of the week in order to maintain fasting effects. For compliance reasons, participants may take one “cheat day” per week at which they follow the fasting rules less strictly. During intermittent fasting, patients are counseled to eat a diet according to the recommendations of the DGE (equivalent to the SD group). Patients additionally attend group sessions every other day during fasting. A physician and a dietician with expertise on fasting monitor these sessions. During the first intensive fasting episode, these sessions are every other day. In the subsequent fasting episodes, the meetings are less frequent (three or four meetings).
- *SD*. Patients are counseled to adhere to a healthy, non-calorie-restricted diet according to the recommendations of the DGE. This diet is predominantly vegetarian with reduced consumption of meat, animal fats, eggs and egg products. Low-fat milk and dairy products are recommended to provide calcium. Dietary recommendations are designed to modify the omega-6 to omega-3 fatty acid ratio to 5:1.

### Adverse events

There are no major risks for patients participating in this study. Minor adverse events of a ketogenic diet and fasting can be headaches, feelings of hunger, fatigue, irritability and dizziness. These side effects are transient and will stop after a few days. Patients will be asked for tolerability of the interventions and any adverse events will be recorded. We do not expect serious adverse events due to our dietary interventions.

### Outcome parameters

Our primary endpoint is the number of new lesions on cranial T2-weighted MRI after 18 months compared to baseline. The secondary MRI endpoint is brain atrophy determined by percentage brain volume change. All MRI scans will be performed in a 3-Tesla MRI scanner (Tim Trio; Siemens, Erlangen, Germany) and will be assessed by an experienced evaluator who is blinded to both clinical data and interventional allocation.

Additional secondary endpoints are the annualized relapse rate, neurological–functional disability progression (EDSS, Multiple Sclerosis Functional Composite), cognition (Symbol Digit Modalities Test), fatigue (Fatigue Severity Scale), depression (Beck Depression Inventory II), muscle strength (handgrip dynamometer), walking endurance (6-min walk test) and quality of life (MSQoL-54). In a subset of patients, we will evaluate glucose variability from continuous glucose measurements over 14 days (FreeStyle Libre Sensors; Abbott). All endpoints will be assessed at baseline, 9 and 18 months by trained and experienced personnel.

For safety monitoring, body weight and composition, dietary intake and vital signs are recorded at regular intervals as well as routine laboratory tests of blood count, kidney and liver parameters in peripheral venous blood. In addition, we want to investigate metabolic, hormonal and immunological effects of the diets. For this, peripheral blood mononuclear cells are isolated for further analysis. In addition, markers of oxidative stress, especially reaction products of anti-oxidative enzymes, are of interest. The polyamine level and cell metabolites will be measured as secondary markers of nutrient availability and autophagy activation in the cell. Stool samples are collected for 16s rRNA sequencing. For a detailed overview of assessments and endpoints, see Table 1.

**Table 1:** SPIRIT flow diagram of the NAMS study

Visit	–1, screening	0, baseline	1, start intervention	2	3	4	5
Month	−4 (max)	−1	0	3	9	15	18
Informed consent	x						
Demographics	x						
Inclusion/exclusion criteria	x	x	x	x	x	x	x
Case history	x						
Medication	x	x	x	x	x	x	x
Vital signs	x			x	x	x	x
Bioelectrical impedance analysis	x				x		x
Anthropometric data	x	x		x	x	x	x
Hand grip strength	x				x		x
Safety laboratory	x			x	x	x	x
Research laboratory		x			x		x
Urine sample	x			x	x	x	x
Stool sample		x			x		x
Multiple Sclerosis Quality of Life-54	x				x		x
Questionnaire on activities	x				x		x
Beck Depression Inventory II	x				x		x
Fatigue Severity Scale	x				x		x
Physical examination	x				x		x
Multiple Sclerosis Functional Composite	x				x		x
Symbol Digit Modalities Test	x				x		x
Six-minute walk test	x				x		x
Expanded Disability Status Scale	x				x		x
Relapse query	x	x	x	x	x	x	x
AE/SAE query		x	x	x	x	x	x
MRI		x			x		x
Four-day food record		x			x		x
Nutritional counseling			x	*	*	*	*

*AE* adverse event, *MRI* magnetic resonance imaging, *SAE* serious adverse event

*There are 10 group sessions for nutritional counseling within 18 months. The fasting diet group has additional meetings during their 7-day-fasts at baseline and after 6 and 12 months

We will inform participants about any conspicuous findings and refer them to their general practitioner for further treatment. After completion of the study, participants have the possibility to be referred to appropriate outpatient nutritional counseling.

### Power calculation

Based on the results of other MS studies in our research center in comparable patient cohorts, an average of three new T2 lesions after 18 months is expected in the SD group. Sample size was calculated with the Wilcoxon (Mann–Whitney) rank-sum test and the significance level was adjusted (Bonferroni) for the two comparisons (KD vs. SD and FD vs. SD). With a sample size of 33 patients per group and a two-sided significance level of 0.025, there is a power of 80% to detect a probability of 0.72 that an observation from group 1 is smaller than that from group 2. This corresponds to a standardized effect size of 0.83 and an expected mean difference in the number of new T2 lesions in each of the intervention groups of 0.5 compared to the SD group after 18 months (Query Advisor 7.0). We expect a dropout rate of approximately 10% and therefore plan to enroll 37 patients per group, 111 patients in total.

### Data management

Each participant will receive a unique identifier upon study entry. This identifier will be used for all data documentation to ensure the participant’s confidentiality. Data will be gathered in source documents and then transferred onto paper case report forms. Later, all data will be digitized and stored in a central database. To improve accuracy of data entry, entries will be verified for proper format and expected range as well as double-checked. Overall data quality will be assured by independent monitoring throughout the study. However, due to the minimal risks of our dietary interventions, a data monitoring committee is deemed unnecessary. Data will be stored for 10 years after study completion and then deleted. Any modification to the current study protocol will be submitted to the institutional review board, all trial participants and the trial investigators.

We only obtain consent for usage of data and samples for the research question described in this protocol. Thus, we do not intend to use participant data or biological samples in ancillary studies.

Results will be personally explained to all study participants, presented on national and international conferences, published in peer-reviewed journals, and disseminated to neurologists and the medical laity. We will comply with the official eligibility guidelines for authorship for all publications and do not intend to use professional writers.

### Data analysis

Confirmatory analysis will be conducted based on the intention-to-treat (ITT) population. The aim is to show that KDs and FDs are superior to the SD, meaning that the number of new T2 lesions after 18 months of dietary intervention adjusted for the baseline value is lower in the KD and FD groups than in the SD group. Although the sample size calculation was conservatively based on the Mann–Whitney *U* test, the final analysis of the primary endpoint will be based on non-parametric analysis of covariance (ANCOVA) on ranks with the treatment group as a fixed factor and adjustment for the baseline number of T2 lesions. The resulting coefficients will be reported with 95% confidence intervals and their corresponding *p*-values compared to a two-sided Bonferroni-adjusted significance level of 0.025 [48].

We do not plan any interim analyses. In the case of more than 5% missing for the primary endpoint, we plan to replace missing values in the outcome parameter using multiple imputation [49] under the missing at random assumption in addition to the complete case analyses.

As a sensitivity analysis, the primary endpoint will be analyzed in the per-protocol population. This population includes all randomized patients who meet the study eligibility criteria and fulfill all compliance criteria throughout the study.

We intend to perform two subgroup analyses, men vs. women and MS medication vs. no MS medication, by adding another main effect for sex or medication, respectively, as well as an interaction term. These subgroup analyses will be analyzed in an exploratory manner to generate possible hypotheses for follow-up studies without adjustment for multiple comparisons. For these, *p*-values may not be interpreted as confirmative.

Safety analysis will include calculation of frequencies and rates of adverse and serious adverse events within 18 months of the interventions.

Secondary endpoints will be analyzed in an explorative manner and descriptives will be given using the mean and standard deviation for sufficiently normal distributed metric variables, the median with limits of the interquartile range (IQR: 25th and 75th percentiles) for skewed metric or ordinal data, as well as absolute and relative frequencies for count data.

## Discussion

To date, this is the first study that investigates long-term efficacy of KDs and FDs on T2 hyperintense lesion load and clinical measures of disease activity and progression in MS patients. This is also the first study that investigates therapeutic effects of repeated prolonged fasting combined with daily intermittent fasting. There are several reasons for the scarcity of such large-scale, long-term studies. The most prominent reasons are, of course, the high demand of financial and human resources. Further, patients often prefer one particular intervention and are therefore prone to withdraw consent when randomized to an undesired intervention. Once patients have committed to such a study, it is challenging to convey dietary interventions in a way that sufficient motivation is aroused. Only this will ensure dietary adherence for the whole intervention period.

There are some critical aspects of our study design. First, we include patients with any MS medication or no medication. From a methodological point of view, including only patients on the same treatment or untreated patients would have been preferable. However, in light of the variety of available treatment options, we would not be able to recruit a sufficient number of patients with the same medication within an acceptable time span. Furthermore, by including patients with different treatments, putative positive results will be more generalizable. Thus, we decided to include all patients who have been stable on or off treatment for at least 6 months. Stratification only differentiates between treatment and no treatment. Having a stratum for every treatment would have resulted in a sample size too unrealistic to recruit and finance. Besides, transferability to clinical routine is only given if the study population, at least in principle, reflects the clinical routine with its diverse application of different treatment options.

Second, a dietary intervention study cannot be blinded completely. Thus, expectations and observer bias cannot be ruled out. However, we try to minimize bias by not communicating any longitudinal data during the study to patients and study personnel in contact with them. Even more important, MRI analysts who evaluate the primary endpoint are blinded for both clinical data and interventional allocation.

Third, we undertake MRI scans of both the cranium and the spinal cord to evaluate disease activity before study entry in order to prevent selection bias. The study MRI, however, only analyzes the cranium, thereby neglecting spinal cord lesions. This entails the risk of overlooking disease activity. However, newly developing spinal cord lesions in MS are often associated with clinical symptoms. Thus, they would be recorded as a relapse within regular clinical assessments. Furthermore, cerebral lesions are more frequent and, therefore, constitute a reliable marker for disease activity.

Fourth, controlling and ensuring adherence to dietary interventions is rather challenging. This is especially true in an outpatient setting during such a long study period. Some patients might be disappointed with their interventional allocation and surreptitiously include some features of the desired group into their diet. This can only be avoided by close and frequent contact between counselor and patient. Furthermore, changing established dietary preferences can be rather challenging and might lead to compliance issues. Patients with suspected or known lack of compliance are excluded from the per-protocol analysis.

One great strength of our study is that patients in the KD group measure their blood ketones at home. Many other studies with low-carbohydrate–high-fat diets did not verify ketosis, which limits the interpretation of results. Although we do not know the actual concentrations of ketone bodies in the brain, blood ketone concentrations have been shown to drive the brain’s overall metabolic rate [50].

Further strengths are the randomized study design, large sample size, long intervention of 18 months and blinded outcome assessment. Moreover, we focus on both MRI-based measures of disease activity and progression and several patient-related endpoints, such as fatigue, depression and quality of life.

Considering epidemiological and preclinical data, on the one hand, and the lack of clinical data, on the other, this study has the potential to provide essential data on the efficacy of KDs and FDs. By analyzing metabolic markers, oxidative stress and the gut microbiome, we hope to shed some light on the mechanisms that underlie these dietary regimens. A better mechanistic understanding might also be applicable to other neurodegenerative diseases.

In conclusion, KDs and FDs have the potential for a safe and inexpensive complementary treatment option in MS and our study might close the gap between promising preclinical data and the lack of clinical data.

## Trial status

This article is based on the study protocol version 1.4 of 23 September 2019. The NAMS started on 24 May 2017. Recruitment will probably continue until March 2020.

## Supplementary information

**Additional file 1.** Approval of institutional review board of Charité - Universitätsmedizin Berlin.

**Additional file 2.** Model informed consent form.

**Additional file 3.** SPIRIT 2013 Checklist: Recommended items to address in a clinical trial protocol and related documents.

## Data Availability

Not applicable.

## Abbreviations

EDSS: Expanded Disability Status Scale
FD: Fasting diet
ICH-GCP: International Conference on Harmonization of Good Clinical Practice
KD: Ketogenic diet
MRI: Magnetic resonance imaging
MS: Multiple sclerosis
NAMS: Nutritional Approaches in Multiple Sclerosis
QoL: Quality of life
RRMS: Relapsing–remitting multiple sclerosis
SD: Standard diet
