# Supplementary material for: Ketogenic diet and fasting diet as Nutritional Approaches in Multiple Sclerosis (NAMS): protocol of a randomized controlled study
Source: Trials. 2020 Jan 2;21:3. doi: 10.1186/s13063-019-3928-9 (PMC6941322; doi:10.1186/s13063-019-3928-9)
Supplement: Supplementary file 2 — Additional file 2. Model informed consent form. [file 13063_2019_3928_MOESM2_ESM.pdf]

Charité | NCRC | AG Klinische Neuroimmunologie | Charitéplatz 1 | 10117 Berlin

Charité – Universitätsmedizin Berlin
Exzellenzcluster NeuroCure
NeuroCure Clinical Research Center
Charitéplatz 1, D-10117 Berlin

AG Klinische Neuroimmunologie
Leitung Prof. Dr. Friedemann Paul
Tel. +49 (0)30 - 450 539 705
Fax +49 (0)30 - 450 539 915
friedemann.paul@charite.de
www.neurocure.de
www.ncrc.de

Einwilligungserklärung zur Teilnahme am wissenschaftlichen Vorhaben

Wirksamkeit ernährungstherapeutischer Ansätze bei Multipler Sklerose

NAMS-Studie

Version 1.3 vom 30.07.2018

Hiermit erkläre ich,

Name: ______________________________

Vorname: ______________________________

Geb. am: ______________________________

Wohnhaft in: ______________________________

dass ich durch Herrn/Frau Dr. ______________________________ ,

NeuroCure Clinical Research Center, Charité – Universitätsmedizin Berlin, Charitéplatz 1, 10117 Berlin

mündlich und schriftlich über das Wesen, die Bedeutung, die Tragweite, den Ablauf und die Risiken der wissenschaftlichen Untersuchung im Rahmen des o.g. wissenschaftlichen Vorhabens, ausführlich und verständlich informiert wurde und ausreichend Gelegenheit hatte, meine Fragen hierzu in einem Gespräch mit einem der beteiligten Ärzte zu klären.

Ich habe insbesondere die mir vorgelegte Studieninformation (Version 1.3 vom 30.07.2018, sowie die ergänzende Information zur Studieninformation gemäß DSGVO (Version 1.0 vom 18.05.2018) verstanden und habe jeweils eine Ausfertigung derselben und dieser Einwilligungserklärung erhalten.

Ich bin bereit, an dieser wissenschaftlichen Untersuchung freiwillig teilzunehmen. Für meine Entscheidung hatte ich ausreichend Zeit.

Einwilligungserklärung zur Datenverarbeitung:

Ich erkläre mich damit einverstanden, dass im Rahmen dieser Studie mich betreffende personenbezogene Daten/Angaben, insbesondere Angaben über meine Gesundheit, durch das Studienteam erhoben und pseudonymisiert in Papierform sowie auf elektronischen Datenträgern aufgezeichnet, verarbeitet und für 10 Jahre gespeichert werden dürfen.

Darüber hinaus bin ich mit der Entnahme, Verschlüsselung, Untersuchung sowie Lagerung (10 Jahre) meiner im Rahmen dieser Studie entnommenen Proben in pseudonymisierter Form für den Zweck der Studie einverstanden.

Ich bin damit einverstanden, dass die im Rahmen dieser Studie entnommenen Proben in verschlüsselter Form (pseudonymisiert) an folgende Labore zum Zweck der o.g. Studie übermittelt werden:

Institut für medizinische Immunologie, AG „Experimentelle Neuroimmunologie“, Charité - Campus Virchow (Augustenburger Platz 1, 13353 Berlin)

Institut für Molekulare Biowissenschaften, Alterung & Zelltod – Labor**,** Universität Graz, Humboldtstrasse 50/EG8010 Graz, Österreich

Endokrinologie, CCR- Center for Cardiovascular Research; Hessische Straße 3-4, 10117 Berlin

Berlin-Brandenburger Centrum für Regenerative Therapien (BCRT), Südstraße 2, 13353 Berlin

Labor Berlin - Charité Vivantes GmbH, Charité Campus Virchow (Sylter Straße 2, 13353 Berlin)

Ferner stimme ich zu, dass meine MRT-Aufnahmen in pseudonymisierter Form einem Neuroradiologen (Charité – Universitätsmedizin Berlin) zur Durchführung einer vorläufigen radiologischen Diagnostik zur Verfügung gestellt werden. Darüber hinaus erkläre ich mich damit einverstanden, dass man mir etwaige Zufallsbefunde, die im Rahmen der MRT-Diagnostik gemacht werden, mitteilt.

Sollte der Studienarzt nicht zugleich der behandelnde Arzt sein, erkläre ich mich weiterhin damit einverstanden, dass der Studienarzt Einsicht in meine Patientenakte erhält. Hierfür entbinde ich den behandelnden Arzt von seiner ärztlichen Schweigepflicht.

Außerdem erkläre ich mich damit einverstanden, dass autorisierte und zur Verschwiegenheit verpflichtete Beauftragte des verantwortlichen Studienleiters (z.B. Monitore) in meine aufbewahrten, personenbezogenen Daten, insbesondere Gesundheitsdaten, Einsicht nehmen dürfen, soweit dies für die Überprüfung der ordnungsgemäßen Durchführung der Studie notwendig ist. Auch hierfür entbinde ich das zuständige Studienpersonal von der ärztlichen Schweigepflicht.

Ich bin auch damit einverstanden, dass die Studienergebnisse in anonymer Form, die keinen Rückschluss auf meine Person zulassen, veröffentlicht werden.

Ich bin über mein Recht zur Auskunft, Berichtigung, Löschung und Sperrung aufgeklärt worden. Mir ist bekannt, dass meine Teilnahme freiwillig ist und ich meine Einwilligung jederzeit ohne Angabe von Gründen und ohne nachteilige Folgen für mich zurückziehen kann und einer Weiterverarbeitung meiner Studiendaten und Proben jederzeit widersprechen und ihre Löschung bzw. Vernichtung verlangen kann. Dabei wird die Rechtmäßigkeit der bis zum Widerruf erfolgten Datenverarbeitung nicht berührt. Nach erfolgtem Widerruf kann ich entscheiden, ob die von mir erhobenen Daten gelöscht werden sollen oder weiterhin für die Zwecke der Studie verwendet werden dürfen.

Ich bin darüber informiert, dass Daten, die bereits in wissenschaftliche Auswertungen eingeflossen sind oder Daten bzw. Proben, die bereits anonymisiert wurden, nicht mehr gelöscht bzw. vernichtet werden können.

Mir ist bekannt, dass ich mich bei Fragen bzgl. der studienbedingten Erhebung meiner personenbezogenen Daten an den verantwortlichen Studienleiter (Prof. Dr. Friedemann Paul; Tel. +49 30 450 539040) **wenden kann. Sollte dieser nicht weiterhelfen können, habe ich die Möglichkeit auch mit der Stabsstelle Datenschutz der Charité - Universitätsmedizin Berlin** (Tel. +49 30 450 580 016 oder E-Mail: datenschutz@charite.de) **Kontakt aufzunehmen. Unabhängig davon, ist mir bekannt, dass es mir freisteht, gerichtliche Hilfe in Anspruch zu nehmen und auch das Recht auf Beschwerde bei einer Aufsichtsbehörde** (Berliner Beauftragte für Datenschutz und Informationsfreiheit: Tel. +49 30 13889-0 oder mail-box@datenschutz-berlin.de) **habe, wenn ich der Ansicht bin, dass die Verarbeitung meiner Daten datenschutzrechtlich nicht zulässig ist.**

<u>Untersuchung von Kurzzeiteffekten des therapeutischen Fastens</u>

(Sub-Kohorte - Optionaler Zusatz für die Teilnehmer alle Gruppen, siehe Erläuterung in der Studieninformation Punkt „Studienablauf“)

Ich bin bereit, an der zusätzlichen Untersuchung teilzunehmen und erkläre mich damit einverstanden, dass meine hierfür gewonnenen Proben und klinische Studiendaten pseudonymisiert an die Universität in British Columbia (Abteilung für Experimentelle Medizin, BC Children's Hospital Research Institute, 950 West 28th Ave, Vancouver, BC **Canada** V5Z 4H4) versendet werden.

Ich wurde aufgeklärt, dass in Drittstaaten (Länder außerhalb der Europäischen Union) andere datenschutzrechtliche Bedingungen gelten können, die mit denen der EU möglicherweise nicht vergleichbar sind. Dennoch soll der Datenschutz nach den gegebenen Möglichkeiten weitestgehend gewährleistet werden.

Ja ☐ Nein ☐ nicht zutreffen ☐

__________________ ____

Berlin, den Unterschrift des/der Teilnehmers/in

Hiermit erkläre ich, den/die o.g. Teilnehmer/in am ____________________ über Wesen, Bedeutung, Tragweite, Ablauf und Risiken des o.g. wissenschaftlichen Vorhabens mündlich und schriftlich aufgeklärt und ihm/ihr eine Ausfertigung der Studieninformation (Version 1.3 vom 30.07.2018), der Ergänzenden Information zur Studieninformation gemäß DSGVO (Version 1.0 vom 18.05.2018) sowie dieser Einwilligungserklärung übergeben zu haben.

__________________ ____

Berlin, den Unterschrift und Name des aufklärenden Arztes
