# Supplementary material for: Ketogenic diet and fasting diet as Nutritional Approaches in Multiple Sclerosis (NAMS): protocol of a randomized controlled study
Source: Trials. 2020 Jan 2;21:3. doi: 10.1186/s13063-019-3928-9 (PMC6941322; doi:10.1186/s13063-019-3928-9)
Supplement: Supplementary file 1 — Additional file 1. Approval of institutional review board of Charité - Universitätsmedizin Berlin. [file 13063_2019_3928_MOESM1_ESM.pdf]

[logo] CHARITÉ

Charité | 10117 Berlin

Prof. Paul Friedemann

Neurology

CCM

Ethics Committee
Ethics Committee I at the Campus Charité Mitte

Chair: Prof. Dr. R. Uebelhack

Secretary: Dr. med. Katja Orzechowski
ethikkommission@charite.de

Address for correspondence:
Charitéplatz 1, 10117 Berlin
Tel: 030/450-517222
Fax: 030/450-517952

http://ethikkommission.charite.de

Cc: Dr. Sandra Lischewski by fax: 539921

Prof. Michalsen, Dr. M. Bock

Date: 09.08.2016

[Efficacy of] Nutritional Approaches in Multiple Sclerosis (NAMS)
Application number: EA1/200/16
Dossier of 09.08.2016 by email

Dear Professor Paul,

We hereby acknowledge receipt of the letter from Dr. Lischewski on 09.08.2016 with the following enclosures:

- Ethics committee application, version from 02.08.2016
- Study information, version 1.1 from 02.08.2016
- Declaration of informed consent, version 1.1 from 02.08.2016

The conditions as per the vote on 06.07.2016 have thus been met. We wish you every success with the performance of the above-mentioned study.

As regards the revision of the declaration of informed consent, the Ethics Committee points out that, according to the information for volunteers, only volunteers who agree to report chance findings are to be included in the study. To avoid misunderstandings and protocol violations, the Ethics Committee therefore recommends deleting from the declaration of informed consent the newly inserted feedback option.

Yours sincerely,

[Signature]

Prof. Dr. med. R. Uebelhack
Committee Chair
